# Interpretable machine learning reveals the association of metabolic, inflammatory, and nutritional indices with ischemic stroke complicated by metabolic abnormality clustering

**DOI:** 10.1186/s12872-026-06443-y

**Published:** 2026-08-13

**Authors:** Shu Chen, Hongyu Chen, Hao Lu, Hao Xu, Da Hu, Kangqin Yang, Yao Jiang

**Affiliations:** 1 Department of Clinical Laboratory Medicine, Suining Central Hospital, No.27 of Dongping North Road, Hedong New District, Suining, Sichuan Province 629000 China; 2 Department of Blood Transfusion, Suining Central Hospital, No.27 of Dongping North Road, Hedong New District, Suining, Sichuan Province 629000 China; 3 Department of Laboratory Medicine, North Sichuan Medical University, No.1 North Medical University Road, Nanchong, Sichuan Province 637000 China; 4 Department of Geriatrics, Suining Central Hospital, No.27 of Dongping North Road, Hedong New District, Suining, Sichuan Province 629000 China

**Keywords:** Acute ischemic stroke, Metabolic abnormality clustering, Machine learning, Clinlabomics model, Metabolic indicators, Risk stratification

## Abstract

**Background:**

This study aimed to explore correlations between metabolic, inflammatory and nutritional composite indices and metabolic abnormality clustering (MAC) in acute ischemic stroke (AIS), and build an interpretable Clinlabomics model to support early risk screening and stratified clinical management.

**Methods:**

This study retrospectively recruited 2,183 AIS patients from Suining Central Hospital between January 2023 and July 2025, who were divided into MAC (*n* = 621) and non-MAC (*n* = 1,562) groups. We included a panel of glucose and lipid metabolic parameters as well as their composite metabolic indices. The cohort was randomly split into a 7:3 training set (*n* = 1,529) and internal test set (*n* = 654), with an independent temporal validation set of 496 patients. In the training cohort, feature selection was performed via least absolute shrinkage and selection operator (LASSO) regression. Eleven machine learning algorithms were used to develop Clinlabomics models. The optimal algorithm was selected based on sensitivity, specificity, accuracy, F1-score, and AUC. Interpretability analysis was performed on the optimal Clinlabomics model using feature importance ranking and SHapley Additive exPlanations (SHAP) to identify core features. Subsequently, univariate and multivariate logistic regression analyses were conducted to identify metabolic signatures significantly associated with the comorbidity of MAC and AIS.

**Results:**

A total of 22 feature variables were identified via LASSO analysis. Eleven machine learning algorithms were adopted to develop Clinlabomics models in the training cohort, among which the recursive partitioning and regression trees (rpart) algorithm was identified as the optimal machine learning algorithm. After hyperparameter tuning, the optimal complexity parameter (cp.) was 0.00015. Following tuning, the AUC values in the training, test, and temporal validation cohorts were as follows: 0.979 (95% CI: 0.970–0.988), 0.968 (95%CI: 0.951–0.985), and temporal validation cohort 0.974 (95% CI: 0.958–0.990). In the temporal validation cohort, this optimal Clinlabomics model correctly identified 129 (84.3%) MAC patients and 337 (98.3%) non-MAC patients. Finally, through integrated analyses, triglyceride–glucose index (TyG), Castelli risk index II (CRI-II), fasting blood glucose (FBG) and atherogenic index of plasma (AIP) were recognized as key metabolic indicators for MAC.

**Conclusions:**

Based on the rpart algorithm, this study developed the optimal clinlabomics model for MAC-AIS comorbidity with satisfactory discriminative ability. TyG, CRI-II, FBG, and AIP were recognized as key metabolic signatures of MAC-AIS comorbidity, providing a reliable tool for early clinical intervention and personalized management.

**Supplementary Information:**

The online version contains supplementary material available at https://doi.org/10.1186/s12872-026-06443-y.

## Introduction

Acute ischemic stroke (AIS) ranks among the leading global causes of mortality and long-term adult disability. Latest data from the Global Burden of Disease (GBD) study demonstrate that population aging and unfavorable lifestyle transitions have driven steady increases in AIS incidence, disability rate and recurrence rate [1, 2]. AIS has complex etiology encompassing vascular risk factors, inflammatory reactions, metabolic derangements, and dysregulated immunity [3]. Although reperfusion regimens and secondary preventive strategies have been progressively optimized, patients with AIS still face prominent risks of unfavorable prognosis. Functional deterioration is especially notable in patients with concomitant metabolic disorders, malnutrition, or systemic inflammation [4]. Among these conditions, metabolic dysfunction has increasingly been recognized as an important contributor to the burden of stroke. It not only accounts for population-level disease-attributable risk but also provides critical evidence for personalized risk stratification [5]. Metabolic abnormality clustering (MAC) exhibits high comorbidity frequency in AIS cohorts and is tightly associated with exacerbated neurological deficits, stroke recurrence and long-term adverse clinical outcomes [6, 7].

MAC refers to a cluster of metabolic disturbances centered on central obesity, hyperglycemia, hypertension, and dyslipidemia, whose pathological essence substantially overlaps with that of metabolic syndrome (MetS) [8]. Among patients with AIS, MAC reflects the coexistence of multiple metabolic and inflammatory disturbances and has been associated with impaired vascular homeostasis, greater neurological injury, and adverse clinical outcomes [9]. Traditional MetS diagnosis relies on binary thresholds (e.g., waist circumference, blood pressure), which tend to mask risk gradients and overlook intra-group heterogeneity. Moreover, retrospective studies often require simplified definitions for key indicators—such as waist circumference—due to missing data, reducing identification sensitivity [8]. Continuous composite metabolic indicators, in contrast, can capture the dynamic spectrum of metabolic disturbances with greater precision, offering higher information density for risk stratification [10].

Composite indices derived from routine laboratory tests have shown considerable potential for characterizing metabolic burden and predicting adverse outcomes in patients with stroke. Metabolic indicators—such as the atherogenic index of plasma (AIP), triglyceride–glucose index (TyG), and METS-IR—capture abnormalities in lipid metabolism and insulin resistance and have been associated with stroke recurrence and functional outcomes [10, 11]. The TyG–AIP product and its longitudinal trajectory may provide additional information beyond individual indicators [12, 13], supporting the evaluation of these continuous metabolic indices as candidate predictors of MAC among patients with AIS. Although inflammation-nutrition composite indicators (e.g., SII, SIRI, HALP) have been linked to adverse stroke outcomes [14], their independent contribution in multivariate models is often masked by metabolic background, with limited incremental predictive efficacy [15, 16]. However, the relative predictive contributions of metabolic and inflammation-related composite indicators remain unclear, particularly among patients with AIS and MAC [12].

Clinlabomics offers a new paradigm for in-depth mining of clinical laboratory data. Proposed by Professor Luo in 2022, this concept integrates multidimensional clinical and laboratory data with machine learning (ML) algorithms to achieve disease identification and risk stratification [17]. Interpretability techniques such as SHAP are applied to decipher the “black box” nature of models and improve model transparency and clinical credibility [18]. Building on this framework, our team has previously constructed interpretable models using laboratory data and successfully identified patients with ischemic stroke combined with hyperuricemia [19], which laid a solid foundation for the subsequent model construction.

Therefore, the present study aims to construct Clinlabomics models based on eleven ML algorithms and identify potential metabolic indicators of comorbid MAC and AIS via interpretable SHAP analysis. By integrating continuous composite metabolic indicators, this strategy was designed to characterize the associations and relative predictive contributions of metabolic, inflammatory, and nutritional indices to MAC among patients with AIS, thereby providing a data-driven basis for the early identification and risk stratification of patients with AIS who are at high risk of MAC.

## Materials and methods

### Study population

**Fig. 1:**
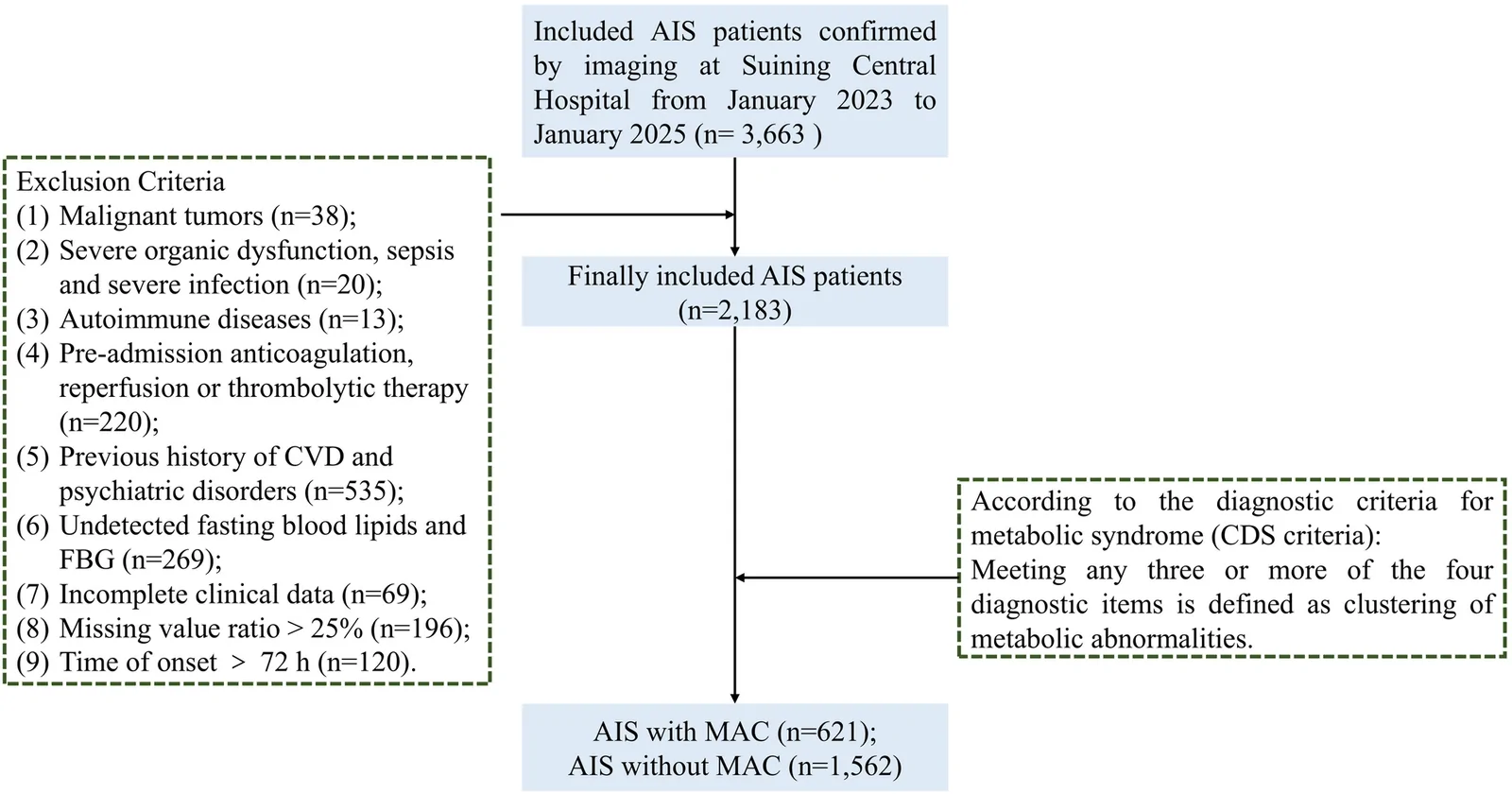
Flowchart of participant screening and grouping for patients with acute ischemic stroke (AIS)

This study retrospectively enrolled patients with IS admitted to the Department of Neurology, Suining Central Hospital, between January 2023 and July 2025. All diagnoses were established according to internationally accepted criteria and confirmed by cranial computed tomography (CT) or magnetic resonance imaging (MRI). Inclusion criteria were: age ≥ 18 years, first-ever ischemic stroke, and complete clinical and laboratory data. Exclusion criteria were: malignant tumors; severe organic dysfunction, sepsis and severe infection; autoimmune diseases; pre-admission anticoagulation, reperfusion or thrombolytic therapy; previous history of CVD and psychiatric disorders; undetected fasting blood lipids and FBG; incomplete clinical data; missing value ratio >25%; time of onset > 72 h. Patients were stratified into the MAC group and the non-MAC group according to the presence of metabolic abnormality clustering, defined using the 2020 Chinese Diabetes Society (CDS) diagnostic criteria for metabolic syndrome. Because waist circumference measurements were not available in our dataset, the abdominal obesity criterion was omitted, and the remaining four metabolic criteria were applied: (1) hyperglycemia: fasting plasma glucose ≥ 6.1 mmol/L, or 2-hour post-load glucose ≥ 7.8 mmol/L, and/or a prior diagnosis of diabetes with ongoing treatment; (2) hypertension: blood pressure ≥ 130/85 mmHg, and/or a prior diagnosis of hypertension with ongoing treatment; (3) hypertriglyceridemia: fasting triglycerides ≥ 1.7 mmol/L; and (4) low high-density lipoprotein cholesterol (HDL-C): fasting HDL-C < 1.04 mmol/L. MAC was defined as the presence of at least three of the above four criteria.This study was approved by the Medical Ethics Committee of Suining Central Hospital (Approval No.: KYLLKS20260044). All participants provided written informed consent at the time of admission. The overall study workflow is illustrated in Fig. 1.

### Data collection

Clinical data and laboratory test results were retrospectively collected through the hospital’s electronic medical record system and laboratory information management system (LIS). Collected data included demographic characteristics (age, gender), medical history (hypertension [HTN], diabetes mellitus [DM], coronary heart disease [CHD], atrial fibrillation [AF]), medication history (antiplatelet therapy [APT], anticoagulant drugs, statin drugs), lifestyle habits (smoking and drinking), time of onset, Trial of Org 10,172 in Acute Stroke Treatment (TOAST) classification, systolic blood pressure (SBP), diastolic blood pressure (DBP), admission National Institutes of Health Stroke Scale (NIHSS) score, discharge modified Rankin Scale (mRS) score, and discharge NIHSS score, as well as laboratory parameters obtained within 24 h of admission, including routine blood counts, electrolytes, liver and kidney function, lipid profiles, and fasting blood glucose. All laboratory tests were performed using a AU5800 automated biochemical analyzer (Beckman, California, USA) to ensure accuracy and consistency. Based on these core indicators, we further calculated a range of composite metabolic, inflammatory, and nutritional indices, including the triglyceride-glucose index (TyG), atherogenic index of plasma (AIP), lipoprotein combine index (LCI), systemic immune-inflammation index (SII), and prognostic nutritional index (PNI). All indices were calculated according to standard methods established in prior literature. The following indices were calculated according to the specified formulas: neutrophil-to-lymphocyte ratio (NLR) = neutrophil count (NEU) / lymphocyte count (LYM) [19]; derived neutrophil-to-lymphocyte ratio (dNLR) = neutrophil count / (white blood cell count − lymphocyte count) [20]; systemic immune-inflammation index (SII) = platelet count (PLT) × NEU / LYM [19]; systemic inflammation response index (SIRI) = NEU × monocyte count (MON) / LYM [19]; pan-immune-inflammation value (PIV) = (neutrophil count × platelet count × monocyte count) / lymphocyte count [20]; hemoglobin, albumin, lymphocyte, and platelet score (HALP) = hemoglobin (HGB) × albumin (ALB) × LYM / PLT [19]; prognostic nutritional index (PNI) = serum albumin (g/L) + 5 × lymphocyte count (×10⁹/L) [21]; triglyceride-glucose index (TyG) = ln [fasting blood glucose (FBG) (mg/dL) × TG (mg/dL) / 2] [19]; atherogenic index of plasma (AIP) = log₁₀(TG / HDL-C) [19]; atherogenic coefficient (AC) = non-HDL-C / HDL-C [22]; lipoprotein combine index (LCI) = TC × TG × LDL-C / HDL-C [19]; Castelli risk index I (CRI-I) = TC / HDL-C [23]; Castelli risk index II (CRI-II) = LDL-C / HDL-C [23]; monocyte-to-albumin ratio (MAR) = monocyte count / serum albumin [24]; uric acid-to-creatinine ratio (UCR) = serum uric acid / serum creatinine; remnant cholesterol (RC) = TC − HDL-C − LDL-C [25]. All index calculations strictly followed standard clinical formulas to ensure scientific validity and comparability.

### Dataset partitioning and cleansing

Prior to dataset partitioning, the dataset was randomly divided into a training set and a test set at a 7:3 ratio with the “createDataPartition” function in the “caret” package. To avoid data leakage, variables with missing values < 25% in the training set were processed by multiple imputation using the “mice” package. The imputation rules generated from the training set were further applied to the test set and validation set without separate imputation. In addition, an additional 496 patients admitted during the same period were independently enrolled as a temporal validation set.

### Feature selection

Least Absolute Shrinkage and Selection Operator (LASSO) regression was implemented for feature selection on the training set, with continuous variables standardized prior to analysis. This penalized regression method was employed to remove redundant variables and identify the optimal feature subset for subsequent model construction.

### Model development and validation

We constructed Clinlabomics models using eleven machine learning (ML) algorithms, including generalized linear model, recursive partitioning and regression trees (rpart), naive Bayes, Bayesian generalized linear model, random forest, extreme gradient boosting (xgbTree), support vector machine with radial basis function kernel, support vector machine with linear kernel, gradient boosting machine, multivariate adaptive regression splines, and elastic net regularized logistic regression. Detailed descriptions of these algorithms and the corresponding hyperparameter tuning procedures are available in our previously published work [19]. In the training dataset, the model demonstrating superior discriminative performance, as reflected by the area under the receiver operating characteristic curve (AUC) and accuracy, was regarded as the optimal model, for which repeated ten-fold cross-validation was adopted to realize hyperparameter optimization. Model discrimination was evaluated using ROC curves; calibration curves were used to assess the goodness-of-fit; and decision curve analysis (DCA) was performed to evaluate the clinical net benefit. The optimal classification threshold was determined by the Youden index based on the training-set ROC curve. Additionally, the Brier score, ranging from 0 to 1, was used to quantify the overall accuracy of predicted probabilities relative to observed outcomes, with lower values indicating better calibration (a value of 0 representing perfect prediction and 1 representing the worst possible calibration) [26]. All model development and validation procedures were performed in the training, test, and temporal validation sets to ensure the robustness and generalizability of the final model.

### Identification of key predictors

Feature importance ranking and SHAP interpretability analysis were applied to quantify the magnitude and direction of the effect of each predictor on model outputs. A Venn diagram was plotted to identify overlapping features derived from feature importance ranking and SHAP analysis, which generated core candidate predictors. These core predictors were then included in univariable and multivariable logistic regression models to screen independent factors related to comorbid MAC and IS. Given that FBG, TG, and HDL-C are important components of MAC diagnostic criteria, we excluded these variables to prevent circular reasoning. We constructed Clinlabomics models based on the remaining variables screened by LASSO regression in the training set to assess the discriminative performance of the optimal ML algorithm for comorbid MAC and IS.

### Statistical analysis

All statistical analyses and model construction were performed using R version 4.3.0. The normality of continuous variables was assessed using the Shapiro–Wilk test. Continuous variables conforming to a normal distribution were presented as means with standard deviations (SD) and compared using the Student’s t-test (or one-way ANOVA for more than two groups); continuous variables with non-normal distributions were presented as medians with interquartile ranges (IQR) and compared using the Mann-Whitney U test. Categorical variables were expressed as counts with percentages and compared using the chi-square test or Fisher’s exact test. We performed univariate and multivariate logistic regression analyses on the screened important variables. The multivariate logistic regression analysis was adjusted for time of onset, age, gender, HTN, CHD, AF, DM, APT, anticoagulant drugs, statin drugs, TOAST classification, smoking status, drinking status, SBP, DBP, admission NIHSS score, discharge mRS score, and discharge NIHSS score. All tests were two-sided, and *P* < 0.05 was considered statistically significant.

## Results

To achieve more accurate diagnosis of MAC, FBG, TC, TG, LDL-C and HDL-C included in this study were complete without missing values. Among the total cohort of 2,183 patients, missing data were observed for Na, K, Cl, WBC, NEU, PLT, HGB, MON, LYM, ALT, AST, TBIL, DBIL, IBIL, Crea, UA and ALB, with missing rates ranging from 0.82% to 1.88%. After multiple imputation using the mice package, no significant differences were detected between the two groups (*P* > 0.05, Supplementary Table 1). A total of 2,183 patients with acute ischemic stroke were included in the study cohort. Based on the presence of MAC, patients were divided into the MAC group (*n* = 621) and the non-MAC group (*n* = 1,562). The overall baseline clinical characteristics of the two groups are compared in Table 1, and the study flowchart is shown in Fig. 1.

**Table 1:** Comparison of baseline clinical characteristics between acute ischemic stroke (AIS) patients with and without MAC

Variable	AIS without MAC (*N* = 1,562)	AIS with MAC (*N* = 621)	*p*-value
Gender (Male, n, %)	899 (58)	366 (59)	0.588
Age (years)	72 (65, 79)	69 (59, 77)	< 0.001
Time of onset	5 (2, 24)	6 (2, 24)	< 0.001
HTN (Yes, n, %)	822 (53)	491 (79)	< 0.001
DM (Yes, n, %)	385 (25)	345 (56)	< 0.001
CHD (Yes, n, %)	134 (9)	55 (9)	0.901
AF (Yes, n, %)	321 (21)	55 (9)	< 0.001
Anticoagulant drugs, n (%)	30 (2)	4 (1)	0.048
APT, n (Yes, %)	19 (1)	4 (1)	0.343
Statin drugs, n (%)	16 (1)	4 (1)	0.554
Smoking (Yes, n, %)	395 (25)	182 (29)	0.062
Drinking (Yes, n, %)	291 (19)	121 (19)	0.689
SBP (mmHg)	144 (126, 161)	150 (134, 166)	< 0.001
DBP (mmHg)	83 (73, 94)	87 (76, 95)	< 0.001
NIHSS at admission	5 (2, 10)	4 (2, 7)	< 0.001
mRS at discharge	2(1, 4)	2 (1, 4)	< 0.001
NIHSS at discharge	4 (1, 8)	3 (1, 6)	< 0.001
Na (mmol/L)	140.0 (138.0, 142.0)	139.7 (137.9, 141.5)	0.037
K (mmol/L)	3.78 (3.50, 4.04)	3.70 (3.46, 3.99)	0.005
Cl (mmol/L)	104.8 (102.2, 107.0)	103.9 (101.5, 106.0)	< 0.001
WBC (10^9^/L)	7.5 (6.0, 9.5)	7.7 (6.2, 9.4)	0.09
NEU (10^9^/L)	5.22 (3.91, 7.36)	5.25 (3.98, 6.98)	0.868
PLT (10^9^/L)	184.5 (143, 231)	194 (150, 239)	0.014
HGB (g/L)	132 (120, 143)	137 (125, 148)	< 0.001
MON(10^9^/L)	0.47 (0.35, 0.61)	0.46 (0.36, 0.63)	0.275
LYM(10^9^/L)	1.29 (0.92, 1.80)	1.50 (1.10, 2.00)	< 0.001
SII	716.26 (425.08, 1259.95)	617.78 (401.09, 1123.76)	0.003
SIRI	1.77 (1.11, 3.30)	1.51 (1.00, 2.89)	0.001
PIV	327.83 (184.59, 629.6)	286.44 (171.16, 550.26)	0.050
NLR	3.96 (2.49, 6.91)	3.34 (2.28, 5.42)	< 0.001
dNLR	2.67 (1.78, 4.40)	2.33 (1.69, 3.49)	< 0.001
HALP	38.29 (26.03, 56.38)	44.48 (29.02, 64.74)	< 0.001
MAR	0.01 (0.01, 0.02)	0.01 (0.01, 0.02)	0.805
FBG (mmol/L)	6.8 (5.7, 8.9)	8.7 (6.9, 12.1)	< 0.001
TyG	8.78 (8.39, 9.18)	9.72 (9.31, 10.28)	< 0.001
ALT (U/L)	18 (13, 25)	20 (15, 29)	< 0.001
AST (U/L)	24 (20, 30)	23 (18, 29)	< 0.001
TBIL (umol/L)	11.80 (8.50, 15.88)	10.60 (7.90, 14.40)	< 0.001
DBIL (umol/L)	2.7 (1.9, 3.8)	2.4 (1.5, 3.3)	< 0.001
IBIL (umol/L)	8.95 (6.30, 12.20)	8.50 (5.80, 11.60)	0.013
Crea (umol/L)	70.9 (58.0, 86.0)	72.3 (59.2, 91.1)	0.031
UA (umol/L)	327.5 (266.0, 396.8)	356.0 (291.0, 431.0)	< 0.001
UA/Cr ratio	4.55 (3.70, 5.55)	4.80 (3.86, 5.96)	< 0.001
ALB (g/L)	40.3 (37.6, 42.9)	41.4 (38.1, 43.8)	< 0.001
TC (mmol/L)	4.83 (4.04, 5.60)	5.11 (4.19, 6.10)	< 0.001
LDL_C (mmol/L)	2.83 (2.27, 3.45)	3.11 (2.48, 3.74)	< 0.001
HDL_C (mmol/L)	1.37 (1.18, 1.62)	1.09 (0.94, 1.35)	< 0.001
TG (mmol/L)	1.13 (0.85, 1.46)	2.26 (1.80, 3.22)	< 0.001
RC	0.5 (0.28, 0.77)	0.76 (0.44, 1.15)	< 0.001
AIP	-0.08 (-0.23, 0.06)	0.29 (0.17, 0.47)	< 0.001
LCI	10.94 (6.19, 18.57)	32.72 (19.12, 55.68)	< 0.001
non_HDL_C (mmol/L)	3.37 (2.71, 4.10)	3.98 (3.14, 4.88)	< 0.001
AC	2.42 (1.87, 3.06)	3.42 (2.77, 4.26)	< 0.001
CRI_I	3.42 (2.87, 4.06)	4.42 (3.77, 5.26)	< 0.001
CRI_II	2.03 (1.59, 2.58)	2.75 (2.25, 3.26)	< 0.001
PNI	47.10 (43.80, 50.65)	49.05 (45.20, 53.10)	< 0.001
TOAST, n (%)			< 0.001
CE	346 (22)	62 (10)	
LAA	437 (28)	208 (33)	
SAO	709 (45)	332 (53)	
SOE	37 (2)	13 (2)	
SUE	33 (2)	6 (1)	

*MAC* Metabolic abnormality clustering, *HTN* Hypertension, *DM* Diabetes mellitus, *CHD* Coronary heart disease, *AF* Atrial fibrillation, *APT* Antiplatelet therapy, *SBP S*ystolic blood pressure, *DBP* Diastolic blood pressure, *NIHSS* National Institutes of Health Stroke Scale, *mRS* Modified Rankin Scale, *WBC* White blood cell, *NEU* Neutrophil, *PLT* Platelet, *HGB* Hemoglobin, *MON* Monocyte, *LYM* Lymphocyte, *SII* Systemic immune-inflammation index, *SIRI* Systemic inflammatory response index, *PIV* Pan-immune-inflammation value, *NLR* Neutrophil-to-lymphocyte ratio, *dNLR* Derived neutrophil-to-lymphocyte ratio, *HALP* Hemoglobin, albumin, lymphocyte, platelet index, *MAR* Monocyte-to-albumin ratio, *FBG* Fasting blood glucose, *TyG* Triglyceride-glucose index, *ALT* Alanine aminotransferase, *AST* Aspartate aminotransferase, *TBIL* Total bilirubin, *DBIL* Direct bilirubin, *IBIL* Indirect bilirubin, Crea, Creatinine, *UA* Uric acid, *ALB* Albumin, *TC* Total cholesterol, *LDL-C* Low-density lipoprotein cholesterol, *HDL-C *High-density lipoprotein cholesterol, *TG* Triglyceride, *RC* Remnant cholesterol, *AIP* Atherogenic index of plasma, *LCI* Lipoprotein combine index, *AC* Atherogenic coefficient, *CRI-I* Castelli risk index I, *CRI-II* Castelli risk index II, *PNI* Prognostic nutritional index, *TOAST* Trial of Org 10,172 in Acute Stroke Treatment, *CE* Cardioembolism, *LAA* Large-artery atherosclerosis, *SAO* Small-artery occlusion, *SOE* Stroke of other determined etiology, *SUE* Stroke of undetermined etiology

Compared with the non-MAC group, the MAC group showed statistically significant differences in age, medical history, blood pressure, stroke severity scores, lipid profiles, glucose, electrolytes, and metabolic, inflammatory, and nutritional composite indices (all *P* < 0.05). The cohort was randomly divided into a training set of 1,529 patients and a test set of 654 patients at a 7:3 ratio, with an additional 496 patients serving as an independent temporal validation set. Baseline characteristics were well-balanced across the training, test, and validation sets (see Supplementary Tables 2–4).

### Construction and validation of the MAC Clinlabomics model

**Fig. 2:**
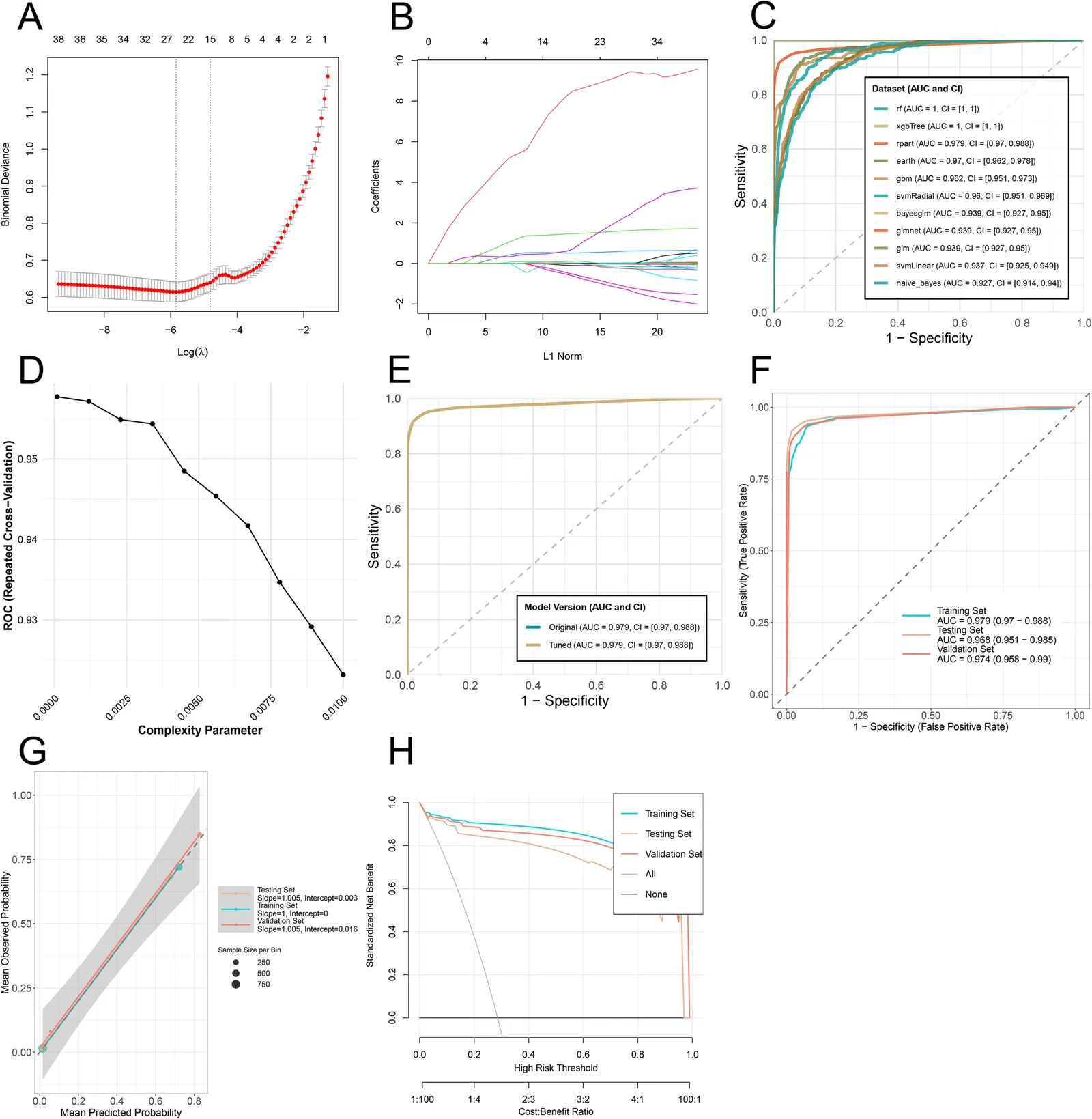
Construction, optimization and performance assessment of the clinical prediction model. **A** LASSO binomial deviance curve with changing log (λ) values for feature screening. **B** LASSO coefficient path plot showing the variation of variable coefficients against L1 norm. **C** ROC curves comparing the discriminative performance of multiple machine learning algorithms. **D** Ten-fold cross-validation curve illustrating the change of model AUC with the complexity parameter (cp.), which guided the selection of the optimal hyperparameter. **E**-**F** ROC curves of the tuned rpart model in the original training set, tuned training set, internal test set and external validation set. **G** Calibration curves of the prediction model across different datasets. **H** Decision curve analysis (DCA) showing the standardized net clinical benefit of the predictive model over a broad range of high-risk thresholds

A total of 38 variables with statistically significant intergroup differences were screened in the training set. Subsequently, these 38 variables were included in the LASSO regression analysis within the training set. The optimal penalty coefficient was determined based on lambda.min obtained via cross-validation. Ultimately, 22 variables retained non-zero regression coefficients (Fig. 2A and B) and were incorporated into the subsequent construction of the prediction model.

A total of 11 machine learning algorithms were employed to construct Clinlabomics models. In the training set, we observed substantial overfitting for the rf and xgbTree algorithms, both of which yielded an AUC of 1. By contrast, the recursive partitioning and regression trees (rpart) algorithm exhibited favorable performance, with a sensitivity of 0.915, specificity of 0.983, accuracy of 0.963, F1-score of 0.935, and an AUC of 0.979 (Supplementary Table 5). Accordingly, the rpart algorithm was selected as the best-performing algorithm to build the final Clinlabomics model (Fig. 2C). Hyperparameter tuning was performed via ten-fold cross-validation; the trend of model AUC as a function of the complexity parameter (cp.) is shown in Fig. 2D, and the optimal cp. value (0.00015) was selected accordingly. The tuned rpart model was subsequently validated in the training set, and the results indicated that the model’s AUC was unaffected before and after hyperparameter tuning (Fig. 2E). The optimal rpart model and corresponding ROC curves for the training (AUC: 0.979, 95% CI: 0.970–0.988), test (AUC: 0.968, 95% CI: 0.951–0.985), and validation sets (AUC: 0.974, 95% CI: 0.958–0.990) is presented in Fig. 2F, indicating excellent discrimination and generalizability. Calibration curves demonstrated good agreement between predicted probabilities and observed outcomes (Fig. 2G). The Brier scores in the training, test, and temporal validation sets were 0.030, 0.049, and 0.039, respectively. Decision curve analysis showed that the model provided substantial clinical net benefit across a wide range of risk thresholds, indicating strong clinical utility (Fig. 2H).

**Fig. 3:**
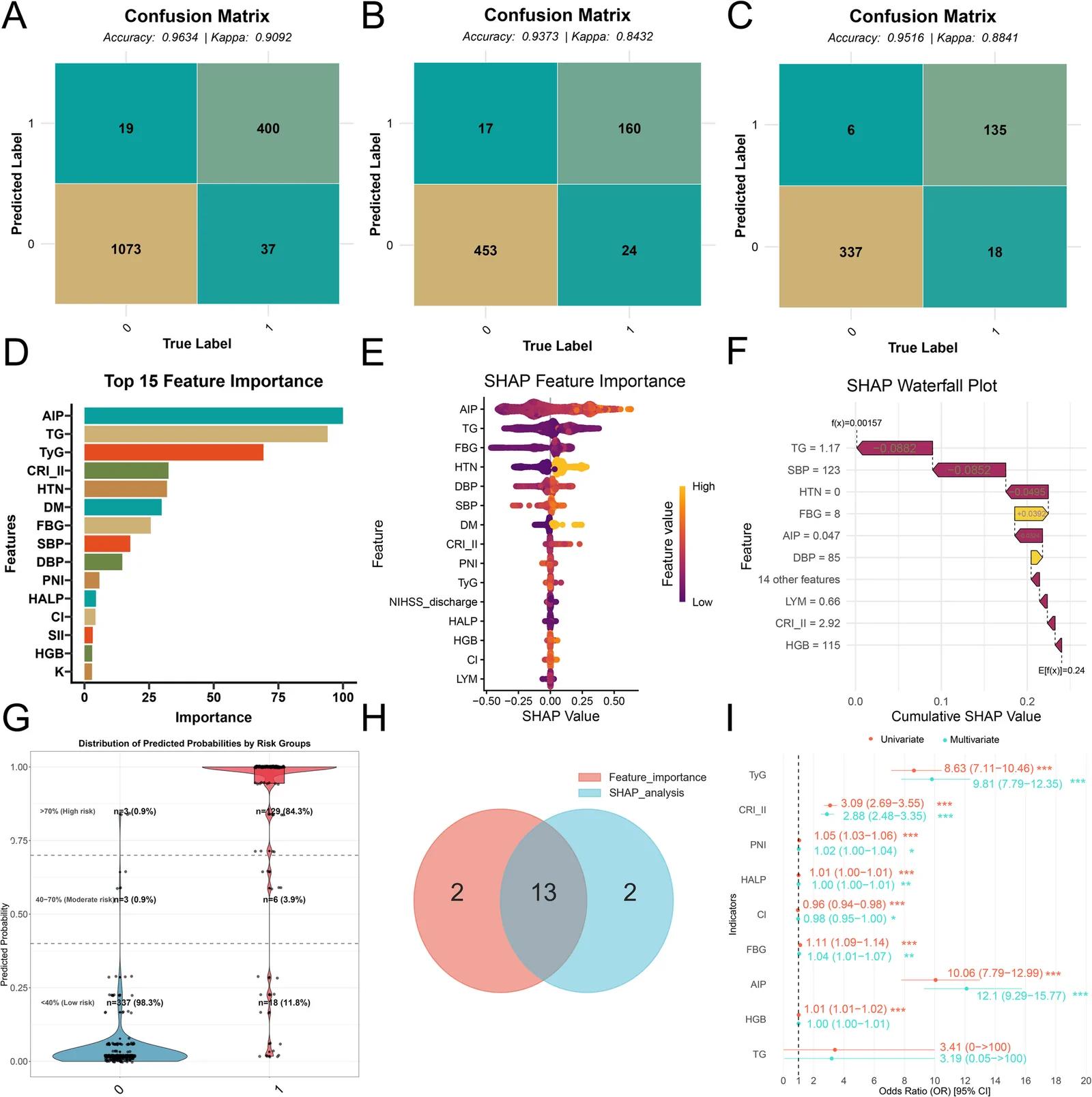
Performance evaluation, feature interpretation and regression analysis of the MAC prediction model. **A**-**C** Confusion matrices of the prediction model in the training set, internal test set, and external validation set. **D** Bar chart of the top 15 predictive variables ranked by model-based feature importance. **E** SHAP summary feature importance plot. **F** SHAP waterfall plot. **G** Violin plot showing the distribution of model-predicted probabilities across predefined low-, moderate-, and high-risk subgroups. **H** Venn diagram. **I** Forest plot

Confusion matrices for the training, test, and validation sets are shown in Fig. 3A and C. The training set achieved an accuracy of 0.9634 (Kappa = 0.9092); the test set, 0.9373 (Kappa = 0.8432); and the validation set, 0.9516 (Kappa = 0.8841). These results indicate stable and reliable classification performance across all datasets.

To further elucidate the model’s key predictors, interpretability analysis was performed. Feature importance ranking revealed that AIP, TG, TyG, and CRI-II were the top four contributors (Fig. 3D). SHAP feature importance analysis and waterfall plots further illustrated the direction and magnitude of each variable’s effect on predictions (Fig. 3E and F). After masking the labels of the temporal validation cohort, we performed risk stratification into low-risk (probability < 40%), moderate-risk (40%–70%), and high-risk (≥ 70%) subgroups. The results demonstrated that the model correctly identified 129 (84.3%) MAC patients and 337 (98.3%) non-MAC patients, indicating that the model effectively discriminates between different risk levels (Fig. 3G). The intersection of feature importance ranking and SHAP analysis results in the Venn diagram identified 13 core indicators (Fig. 3H), including TyG, CRI-II, PNI, HALP, SBP, Cl-, HTN, DBP, FBG, DM, AIP, HGB, and TG. Among them, TyG, CRI-II, FBG, AIP, and TG are metabolic indicators closely associated with MAC. Considering that FBG and TG incorporated into the initial model were strongly associated with the diagnosis of MAC, we excluded these two indicators and reconstructed an rpart model based on the remaining variables for sensitivity analysis. The newly established model maintained favorable predictive performance (sensitivity: 0.883; specificity: 0.946; accuracy: 0.928; F1-score: 0.875; AUC: 0.960, Supplementary Fig. 1A). After hyperparameter tuning, the optimal cp. of the model was determined as 0.00134 (Supplementary Fig. 1B), and no significant difference in model AUC was observed before and after hyperparameter optimization (Supplementary Fig. 1C). The AUC values of ROC curves for the training set, test set and temporal validation set were 0.960 (95% CI: 0.950–0.971), 0.912 (95% CI: 0.886–0.939), and 0.948 (95% CI: 0.928–0.969, Supplementary Fig. 1D), respectively. Calibration curves (Supplementary Fig. 1E) and DCA (Supplementary Fig. 1F) corresponding to the three datasets demonstrated that the model achieved satisfactory calibration and yielded stable net clinical benefit. The Brier scores of the three cohorts were 0.059, 0.106, and 0.073, respectively. Kappa values calculated from confusion matrices for the model across the three datasets were 0.8213, 0.6737, and 0.785 (Supplementary Fig. 1G–I). In summary, the reconstructed rpart model after removing FBG and TG (factors closely linked to MAC diagnosis) retained robust predictive capacity. This approach effectively avoided circular reasoning and thoroughly verified the stability of the model.

### Univariable and multivariable analysis of MAC in ischemic stroke

Univariable logistic regression analysis of the 13 core indicators identified by the Venn diagram showed that AIP, TyG, CRI-II, FBG, HALP, Cl^-^, and PNI were significantly associated with MAC risk (all *P* < 0.05). As shown in Fig. 3I, in the multivariable analysis, AIP (OR = 12.10, 95% CI: 9.29–15.77), TyG (OR = 9.81, 95% CI: 7.79–12.35), CRI-II (OR = 2.88, 95% CI: 2.48–3.35), and FBG (OR = 1.04, 95% CI: 1.01–1.07) emerged as independent risk factors, while chloride (Cl^⁻^) (OR = 0.98, 95% CI: 0.95–1.00) was identified as an independent protective factor for MAC (all *P* < 0.05).

## Discussion

In the present study, we developed a Clinlabomics model for MAC based on the optimal rpart algorithm, which exhibited favorable predictive performance. Integrating feature importance ranking, SHAP analysis, and univariate and multivariate logistic regression, we identified TyG, CRI-II, FBG and AIP as key metabolic indicators for MAC. This study offers evidence-based laboratory evidence for early identification, risk stratification, and individualized intervention in ischemic stroke combined with metabolic abnormality clustering.

First, we constructed Clinlabomics models for patients with MAC and AIS using 11 ML algorithms and identified the optimal rpart algorithm. Clinlabomics models are valuable for disease early warning and prognostic prediction [17]. Our prior works established Clinlabomics models for patients with comorbid IS and HUA, as well as comorbid IS and DM, which achieved favorable predictive performance with AUC values > 0.80 across multiple datasets. Furthermore, we uncovered distinct metabolic signature profiles for the two comorbid conditions [19, 23]. These results reveal divergent metabolic disturbance patterns among different comorbidities and unique laboratory metabolic biomarkers of stroke-related complications, validating the capacity of Clinlabomics to distinguish various disease phenotypes. Similarly, the present study built a Clinlabomics model for comorbid MAC and AIS. The model yielded AUC values over 0.95 in three datasets, exhibiting excellent and stable performance to screen high-risk patients with MAC combined with AIS. In addition, via integrating feature importance analysis, SHAP interpretability analysis, and univariate and multivariate logistic regression analyses, we identified TyG, CRI-II, FBG and AIP as the specific metabolic signature profile for comorbidity of MAC and AIS. Regarding the optimal algorithm, rpart (recursive partitioning and regression trees) was identified as the best-performing model among the 11 machine learning algorithms compared in this study. The rpart algorithm constructs a decision tree based on recursive partitioning, which iteratively selects clinical laboratory indicators that maximize between-group differences while minimizing within-group variation, thereby precisely mining the associations between indicators and disease status while balancing predictive accuracy and interpretability. Decision-tree-based methods have been widely applied in clinical prediction research, demonstrating excellent stability and predictive performance across various disease risk stratification and prognostic prediction tasks, with the capacity to handle complex nonlinear relationships in clinical data [27, 28]. Compared with other machine learning algorithms, rpart requires no complex data preprocessing and is highly tolerant of missing values and outliers; moreover, its tree structure is intuitive and easy to understand, facilitating clinical translation. In addition, overfitting can be effectively avoided through pruning, thereby enhancing generalizability—a key reason for the excellent performance achieved in this study.

Among these predictors, AIP, a composite indicator integrating the pro-atherogenic (TG) and anti-atherogenic (HDL-C) dimensions, was identified as one of the most valuable metabolic predictors of MAC in this study. Notably, beyond AIP, other routine laboratory-derived composite indices have also demonstrated utility in identifying metabolic abnormalities; for example, Marra et al. [29] reported that CBC-derived inflammation indexes were useful in predicting metabolic syndrome in adults with severe obesity, supporting the clinical value of accessible laboratory parameters for metabolic risk screening. AIP not only quantifies lipid imbalance but also reflects a deeper linkage with the inflammation-oxidative stress network. Bazyar et al. [22] showed in a double-blind, placebo-controlled trial of patients with type 2 diabetes that the reduction in AIP following rutin intervention was accompanied by concurrent decreases in IL-6 and MDA together with a significant increase in HDL-C. Consistent with these biological mechanisms, accumulating clinical evidence has also supported the prognostic value of AIP in AIS. The clinical predictive value of AIP in AIS has been validated across multiple studies. Liu et al. [30] reported in a prospective cohort of 1,463 AIS patients that elevated baseline AIP was independently associated with 3-month poor functional outcome (OR = 1.84, 95% CI: 1.23–2.53), with a stronger association in the large artery atherosclerosis (LAA) subtype (OR = 1.90, 95% CI: 1.53–2.62). Wang et al. [31] further found that AIP was significantly associated with early neurological deterioration in AIS patients (OR = 3.26, 95% CI: 1.49–7.13).

Notably, the association between AIP and MAC itself has also been directly documented. In the NHANES cohort (2013–2020), Sang et al. [32] demonstrated that each 1-SD increase in AIP corresponded to markedly higher odds of metabolic syndrome (OR = 10.9, *P* < 0.001), and in CHARLS, AIP mediated 62.9% of the association between pro-inflammatory diet and MetS. A systematic review encompassing 197,279 patients further confirmed that elevated AIP consistently predicts major adverse cardiovascular events (HR 1.07–1.19), and cumulative AIP exposure substantially increases stroke risk (HR = 2.49) in patients with MetS [33]. More recently, Xue et al. [34] in 346,868 UK Biobank participants with cardiovascular-kidney-metabolic (CKM) syndrome stages 0–3, found that AIP-related indices were independently associated with the incidence and progression of cardiometabolic multimorbidity. Given that dyslipidemia is a core component shared by MAC and its downstream atherosclerotic complications, AIP may serve as a crucial link bridging lipid imbalance and MAC. These findings are consistent with our results, in which AIP emerged as one of the most important predictors of MAC.

In addition to AIP, TyG serves as a credible surrogate marker for insulin resistance and bears predictive effects independent of classic obesity indicators including BMI and waist circumference. Among patients with AIS, insulin resistance accelerates thrombosis and promotes atherosclerosis, thereby worsening stroke severity and long-term prognosis [35]. Miao et al. [36] studied 1,523 patients with ischemic stroke and verified that high TyG index was independently associated with increased common carotid artery intima–media thickness (OR = 1.56, 95% CI: 1.06–2.28). This indicated that insulin resistance deteriorates stroke outcomes by promoting large artery atherosclerotic progression. These findings suggest that insulin resistance may contribute to ischemic stroke through the progression of large-artery atherosclerosis. In another cohort of 1,970 non-diabetic individuals with small-vessel occlusive stroke, Wu et al. [37] found that participants in the top TyG quartile had substantially higher 1-year stroke recurrence risk (Q4 vs. Q1: OR = 3.10, 95% CI: 1.37–8.02).

Notably, continuous TyG can accurately reflect varying degrees of insulin resistance, while binary diagnosis of diabetes fails to depict the full dynamic range of metabolic disorders. Based on the MIMIC-IV database, Cai et al. [38] demonstrated that TyG independently predicted in-hospital death (HR = 1.37, 95% CI: 1.05–1.78) and ICU mortality (HR = 1.65, 95% CI: 1.24–2.20) in critically ill stroke patients. Beyond its role in stroke, TyG has been longitudinally linked to the development of MAC itself. In a prospective cohort of 1,129 adults free of metabolic syndrome at baseline, Bennouar et al. showed that TyG-related indices, particularly TyG-WC, significantly predicted incident metabolic syndrome during a median 45-month follow-up (aHR = 1.44 per SD) [39]. More importantly, recent studies have linked TyG-related indices with the broader cardiovascular-kidney-metabolic (CKM) syndrome, a conceptual framework closely related to metabolic abnormality clustering. Li et al. [40] demonstrated in a nationwide prospective cohort that elevated TyG-BMI was significantly associated with future cardiovascular disease risk in populations with CKM syndrome stages 0–3 (HR = 1.31, 95% CI: 1.24–1.38). Lu et al. [41] further reported that cumulative changes in the TyG index were independently associated with stroke incidence in individuals with CKM syndrome, and Yue et al. [42] confirmed the association of novel TyG-related indices with incident stroke in early-stage CKM syndrome. Collectively, these findings indicate that TyG-related markers capture clusters of metabolic abnormalities beyond isolated glycemic disturbances, supporting TyG as a powerful metabolic predictive marker linking insulin resistance, metabolic clustering, and ischemic stroke in our study.

Among the core predictors, CRI-II and FBG further complemented the metabolic signature of MAC in patients with AIS. CRI-II, defined as the LDL-C/HDL-C ratio, is a classical atherogenic lipid index that reflects the balance between pro-atherogenic and anti-atherogenic lipoproteins. Recent evidence has demonstrated that CRI-II is closely associated with carotid atherosclerosis and intraplaque neovascularization in patients with carotid stenosis (OR = 1.57, 95% CI: 1.05–2.35) [43], and traditional lipid ratios including CRI-II have been shown to correlate with carotid atherosclerosis burden in patients with ischemic stroke (Q4 vs. Q1: OR = 1.48, 95% CI: 1.03–2.14) [44], supporting its role in the atherosclerotic processes underlying MAC. In our previous Clinlabomics studies, lipid-related composite indices also emerged as key discriminators of stroke-related comorbidities, including ischemic stroke complicated by hyperuricemia and diabetes mellitus [19, 23], further corroborating the value of lipid ratios in metabolic stratification. FBG, as a core component of the MAC diagnostic criteria, reflects the glycemic dimension of metabolic dysfunction. Although the diagnostic overlap between FBG and MAC was carefully addressed in our feature selection strategy (as detailed in the Methods), FBG remained independently associated with MAC after adjustment, consistent with the well-established role of hyperglycemia in vascular disease risk (HR = 2.27, 95% CI: 1.95–2.65) [45] and with our previous finding that glucose-related markers are among the top discriminators for ischemic stroke complicated by diabetes [23]. Taken together, CRI-II and FBG capture the lipid and glycemic dimensions of metabolic abnormality clustering, respectively, and together with AIP and TyG constitute a comprehensive, routine-laboratory-based metabolic panel for MAC identification.

Several limitations of the present study need to be acknowledged. First, all participants were enrolled from a single-center retrospective cohort, raising the possibility of selection bias. Second, waist circumference records were missing in our dataset. Accordingly, MAC was diagnosed with revised criteria that excluded the central obesity index, which might result in missed diagnoses for patients with central obesity-dominant metabolic disorders. Subsequent prospective trials need to include waist circumference measurements to validate the integrity of the MAC diagnostic system. Third, this analysis focused solely on cross-sectional correlations of baseline laboratory indicators. Dynamic fluctuations of biomarkers after hospitalization and the modifying effects of clinical treatments on MAC were not incorporated into our analysis.

## Conclusions

This study, using interpretable machine learning algorithms, demonstrates that metabolic indices, including TyG, CRI-II, FBG, and AIP are closely associated with MAC in patients with AIS. The rpart-based prediction model exhibits excellent discriminative performance (AUC = 0.979, 0.968, and 0.974) with robust internal and temporal validation. The model efficiently identifies core predictors and enables accurate identification and risk stratification of this comorbid condition, providing a practical tool for early clinical intervention and personalized management. Future multicenter prospective studies are warranted to further validate and optimize the model.

## Supplementary Information

Supplementary Material 1.

Supplementary Material 2.

Supplementary Material 3.

Supplementary Material 4.

Supplementary Material 5.

Supplementary Material 6.

## Data Availability

All data generated or analysed during this study are included in this published article.

## References

[1] Cercel A, Mihaela A, Doeppner TR, Hermann DM, Martin L, Popa-Wagner A. Ischemic stroke and comorbidities: Impact on recovery and the role of genetics and epigenetics [J]. Mech Ageing Dev. 2026;229:112127. 10.1016/j.mad.2025.112127 41276229

[2] Fan J, Li X, Yu X, Liu Z, Jiang Y, Fang Y, et al. Global Burden, Risk Factor Analysis, and Prediction Study of Ischemic Stroke, 1990–2030 [J]. Neurology. 2023;101(2):e137–50. 10.1212/WNL.0000000000207387 PMC10351546 37197995

[3] Lei W, Zhuang H, Huang W, Sun J. Neuroinflammation and energy metabolism: a dual perspective on ischemic stroke [J]. J Transl Med. 2025;23(1):413. 10.1186/s12967-025-06440-3 PMC11983748 40211331

[4] Li J, Yang J. Development and validation of a novel nutrition-inflammation prognostic score for predicting 30-day mortality in critically ill stroke patients [J]. Front Nutr. 2025;12:1658896. 10.3389/fnut.2025.1658896 PMC12442555 40969611

[5] Xiao Z, Deng L, Duan Y, Wang B, Liu A. Burden trajectories of stroke attributable to lipid-glucose dysfunction and biomarker validation: evidence from the global burden of disease (GBD) 2023 and the China health and retirement longitudinal study (CHARLS) [J]. Cardiovasc Diabetol. 2026;1–33. 10.1186/s12933-026-03269-y PMC13551825 42324526

[6] Zhang YN, Wu Q, Cui Y, Zhang NN, Chen HS. Metabolic Syndrome and Efficacy of Remote Ischemic Postconditioning in Acute Moderate Ischemic Stroke: A Post Hoc Analysis of the RICAMIS Trial [J]. J Am Heart Assoc. 2025;14(6):e037859. 10.1161/JAHA.124.037859 PMC12132609 40079308

[7] Duan Z, Wei X, Liu H, Zhai Y, Hu T, Xu J, et al. The effect of metabolic syndrome and/or hyperglycemia on outcomes of acute ischemic stroke patients treated with intravenous thrombolysis [J]. Int J Stroke. 2022;17474930211067352. 10.1177/17474930211067352 35255756

[8] Wilkie J, Huang XF, Wang L, Win KT. Clustering beyond the binary: Stable machine-learning phenotypes of metabolic syndrome [J]. Comput Methods Programs Biomed. 2026;285:109538. 10.1016/j.cmpb.2026.109538 42424777

[9] Jin Z, Chen Q, Zhou L, Ye K, Liu Z, Jiang W, et al. Integrated Metabolic and Inflammatory Clustering Reveals Distinct Risk Profiles for Digestive Diseases [J]. Adv Sci (Weinh). 2025;12(44):e11000. 10.1002/advs.202511000 PMC12667462 41017045

[10] Wang T, Yi Z, Tan Y, Huang Y, Li T, Gao S, et al. Changes in the metabolic score for insulin resistance index for risk prediction of stroke in middle-aged and older Chinese population [J]. Epma j. 2024;15(4):599–610. 10.1007/s13167-024-00388-y PMC11612103 39635019

[11] He Y, Xu F, Deng J, Li M, Leng S. Association of Composite Metabolic Indices With Incident Carotid Plaque: A Chinese Cohort Study [J]. J Am Heart Assoc. 2026;15(6):e047902. 10.1161/JAHA.125.047902 PMC13055790 41823235

[12] Yang D, Deng Y, Su Z, Zhao M. Association of C-reactive protein-triglyceride glucose index and its modified indices with stroke risk in individuals at cardiovascular-kidney-metabolic syndrome stages 0–3: a population-based cohort study [J]. Cardiovasc Diabetol. 2026;1–53. 10.1186/s12933-026-03282-1 PMC13491843 42415086

[13] Zhou Z, Yang J, Hou J, Zhang L. The predictive value of C-reactive protein-triglycerides-glucose index-waist-to-height ratio for stroke: a nationwide cohort study [J]. Cardiovasc Diabetol. 2026;25(1):18. 10.1186/s12933-025-03065-0 PMC12817842 41547799

[14] Jia B, Ma R, Zhu P, Zhang R, Li S, Jiang Z. Combined nutritional and inflammatory risk stratification for predicting functional outcomes in acute ischemic stroke [J]. Front Nutr. 2026;13:1869783. 10.3389/fnut.2026.1869783 PMC13385485 42483645

[15] Liu X, Zheng X, Chen Z, Chen H, Xie M, Zhang J. Associations of inflammation-related nutritional and metabolic status indices CAR and CTI with 90-day unfavorable functional outcomes in patients with acute ischemic stroke [J]. Front Nutr. 2026;13:1790922. 10.3389/fnut.2026.1790922 PMC13050733 41948290

[16] Mahapatro A, Jala SRA, Ghodous S, Rashidian P, Mohanty E, Mirchandani M, et al. Systemic immune-inflammation index as a novel biomarker for metabolic syndrome: A systematic review and meta-analysis [J]. Med (Baltim). 2026;105(27):e49667. 10.1097/MD.0000000000049667 PMC13337060 42410855

[17] Wen X, Leng P, Wang J, Yang G, Zu R, Jia X, et al. Clinlabomics: leveraging clinical laboratory data by data mining strategies [J]. BMC Bioinform. 2022;23(1):387. 10.1186/s12859-022-04926-1 PMC9509545 36153474

[18] Tang X, Tang M, Liu W, Cui S. Explainable machine learning for stroke risk prediction: a comparative study with SHAP-based interpretation. Front Neurol. 2026;16:1716984. 10.3389/fneur.2025.1716984 PMC12832496 41602961

[19] Jiang Y, Li Q, Hu D, Liao H, Chen S, Xu H, et al. Identifying metabolic parameters as key indicators of hyperuricemia and ischemic stroke comorbidity via interpretable Clinlabomics models [J]. Front Endocrinol (Lausanne). 2025;16:1737419. 10.3389/fendo.2025.1737419 PMC12834788 41607461

[20] Zhang J, Wang Y, Liu H, Lei Z, Cheng S, Cao H. The association between eight complete blood count-derived inflammatory markers and muscle health [J]. Front Nutr. 2025;12:1498757. 10.3389/fnut.2025.1498757 PMC11830586 39963665

[21] Kumar A, Gurram L, Naga Ch P, Nayak P, Mulye G, Chopra S, et al. Correlation of Hematological Parameters With Clinical Outcomes in Cervical Cancer Patients Treated With Radical Radio(chemo)therapy: A Retrospective Study [J]. Int J Radiat Oncol Biol Phys. 2024;118(1):182–91. 10.1016/j.ijrobp.2023.07.022 37506980

[22] Bazyar H, Moradi L, Zaman F, Zare Javid A. The effects of rutin flavonoid supplement on glycemic status, lipid profile, atherogenic index of plasma, brain-derived neurotrophic factor (BDNF), some serum inflammatory, and oxidative stress factors in patients with type 2 diabetes mellitus: A double-blind, placebo-controlled trial [J]. Phytother Res. 2023;37(1):271–84. 10.1002/ptr.7611 36101997

[23] Jiang Y, Qian A, Chen S, Wu Q, Xu H, Zheng C, et al. Identification of key metabolic indicators associated with the comorbidity of ischemic stroke and diabetes mellitus using an optimal interpretable clinlabomics model [J]. Front Cardiovasc Med. 2026;13:1874711. 10.3389/fcvm.2026.1874711 PMC13341514 42422181

[24] Li H, He Y, Chen B, Liu Z, Zhu Z, Zhu W, et al. Associations of inflammation/nutrition-related indicators (RAR and MAR) with chronic kidney disease: evidence from NHANES 2005–2018 [J]. Front Nutr. 2026;13:1725996. 10.3389/fnut.2026.1725996 PMC13159209 42125316

[25] Fu L, Tai S, Sun J, Zhang N, Zhou Y, Xing Z, et al. Remnant Cholesterol and Its Visit-to-Visit Variability Predict Cardiovascular Outcomes in Patients With Type 2 Diabetes: Findings From the ACCORD Cohort [J]. Diabetes Care. 2022;45(9):2136–43. 10.2337/dc21-2511 PMC9472497 35834242

[26] Luo X, Li B, Zhu R, Tai Y, Wang Z, He Q, et al. Development and validation of an interpretable machine learning model for predicting in-hospital mortality for ischemic stroke patients in ICU [J]. Int J Med Inf. 2025;198:105874. 10.1016/j.ijmedinf.2025.105874 40073651

[27] Xing Y, Jin Y, Liu Y. Construction and comparison of short-term prognosis prediction model based on machine learning in acute ischemic stroke [J]. Heliyon. 2024;10(2):e24232. 10.1016/j.heliyon.2024.e24232 PMC10792580 38234895

[28] He R, Zhang K, Li H, Gu M. Development and validation of inpatient mortality prediction models for patients with hyperglycemic crisis using machine learning approaches [J]. BMC Endocr Disord. 2025;25(1):76. 10.1186/s12902-025-01873-9 PMC11948940 40140995

[29] Marra A, Bondesan A, Caroli D, Sartorio A. Complete Blood Count (CBC)-Derived Inflammation Indexes Are Useful in Predicting Metabolic Syndrome in Adults with Severe Obesity [J]. J Clin Med. 2024;13(5):1–15. 10.3390/jcm13051353 PMC10932131 38592161

[30] Liu H, Liu K, Pei L, Li S, Zhao J, Zhang K, et al. Atherogenic Index of Plasma Predicts Outcomes in Acute Ischemic Stroke [J]. Front Neurol. 2021;12:741754. 10.3389/fneur.2021.741754 PMC8542679 34707558

[31] Wang Q, Jiang G, Yan L, Chen R, Liu Y, Liu L, et al. Association of atherogenic index of plasma with early neurological deterioration in patients with acute ischemic stroke [J]. Clin Neurol Neurosurg. 2023;234:108014. 10.1016/j.clineuro.2023.108014 37857235

[32] Sang J, Zhang K, Kan C, Li X, Qiu H, Guo Z, et al. The Mediating Role of Atherogenic Index of Plasma in the Association Between Dietary Inflammatory Index and Metabolic Syndrome: Evidence from NHANES and CHARLS [J]. Metab Syndr Relat Disord. 2026;15578518261472829. 10.1177/15578518261472829 42544604

[33] Shaybah ME, Alenezi GT, Alqahtani MM, Yamaniey AS, Al Farhan HH, Muslmani AK, et al. Effectiveness of plasma atherogenic index as a predictor of cardiovascular risk in patients with metabolic syndrome and related cardiometabolic conditions: a systematic review [J]. Cardiovasc Endocrinol Metabol. 2026;15(e00361-3):1–10. 10.1097/XCE.0000000000000361 PMC13327390 42396551

[34] Xue F, Han Q, Li Y, Cheng C, Xie L, Yang J, et al. Associations of the atherogenic index of plasma and its modified indices with the incidence and progression of cardiometabolic multimorbidity in individuals with cardiovascular-kidney-metabolic syndrome stages 0–3: a prospective cohort study [J]. Cardiovasc Diabetol. 2026;1–49. 10.1186/s12933-026-03255-4 PMC13491610 42289689

[35] Ding PF, Zhang HS, Wang J, Gao YY, Mao JN, Hang CH, et al. Insulin resistance in ischemic stroke: Mechanisms and therapeutic approaches [J]. Front Endocrinol (Lausanne). 2022;13:1092431. 10.3389/fendo.2022.1092431 PMC9798125 36589857

[36] Miao M, Zhou G, Bao A, Sun Y, Du H, Song L, et al. Triglyceride-glucose index and common carotid artery intima-media thickness in patients with ischemic stroke [J]. Cardiovasc Diabetol. 2022;21(1):43. 10.1186/s12933-022-01472-1 PMC8933990 35303881

[37] Wu L, Zhu J, Li C, Zhu J, Dai Z, Jiang Y, et al. Association of triglyceride-glucose index with ischemic stroke recurrence in nondiabetic patients with small vessel occlusion: a multicenter hospital-based prospective cohort study [J]. Cardiovasc Diabetol. 2022;21(1):250. 10.1186/s12933-022-01693-4 PMC9673408 36397084

[38] Cai W, Xu J, Wu X, Chen Z, Zeng L, Song X, et al. Association between triglyceride-glucose index and all-cause mortality in critically ill patients with ischemic stroke: analysis of the MIMIC-IV database [J]. Cardiovasc Diabetol. 2023;22(1):138. 10.1186/s12933-023-01864-x PMC10262584 37312120

[39] Bennouar S, Bachir Cherif A, Fissah M, Abdi S. Longitudinal comparative performance of triglyceride-glucose-related indices and estimated glucose disposal rate for predicting metabolic syndrome risk [J]. J Diabetes Metab Disord. 2026;25(2):215. 10.1007/s40200-026-02021-3 PMC13407780 42523848

[40] Li W, Shen C, Kong W, Zhou X, Fan H, Zhang Y, et al. Association between the triglyceride glucose-body mass index and future cardiovascular disease risk in a population with Cardiovascular-Kidney-Metabolic syndrome stage 0–3: a nationwide prospective cohort study [J]. Cardiovasc Diabetol. 2024;23(1):1–11. 10.1186/s12933-024-02352-6 PMC11308445 39113004

[41] Lu L, Chen Y, Liu B, Li X, Wang J, Nie Z, et al. Association between cumulative changes of the triglyceride glucose index and incidence of stroke in a population with cardiovascular-kidney-metabolic syndrome stage 0–3: a nationwide prospective cohort study [J]. Cardiovasc Diabetol. 2025;24(1):202. 10.1186/s12933-025-02754-0 PMC12070779 40355933

[42] Yue Y, Li P, Sun Z, Murayama R, Li Z, Hashimoto K, et al. Association of novel triglyceride-glucose-related indices with incident stroke in early-stage cardiovascular-kidney-metabolic syndrome [J]. Cardiovasc Diabetol. 2025;24(1):301. 10.1186/s12933-025-02854-x PMC12291248 40707904

[43] Cui L, Zhou F, Chen Y, Liu R, Xing Y. The lipid ratio castelli’s risk index II is a novel biomarker for intraplaque neovascularization in patients with carotid stenosis [J]. Lipids Health Dis. 2025;24(1):1–10. 10.1186/s12944-025-02821-1 PMC12797835 41353547

[44] Miao M, Ye C, Wang Z, Long J, You S, Sun Y, et al. Traditional Lipid Ratios and Carotid Atherosclerosis in Patients with Ischemic Stroke [J]. Cerebrovasc Dis. 2026;55(1):103–13. 10.1159/000545676 40273898

[45] Emerging Risk Factors Collaboration, Sarwar N, Gao P, Seshasai SR, Gobin R, Kaptoge S, Di Angelantonio E et al. Diabetes mellitus, fasting blood glucose concentration, and risk of vascular disease: a collaborative meta-analysis of 102 prospective studies [J]. Lancet. 2010;375(9733):2215–2222. Erratum in: Lancet. 2010;376(9745):958. 10.1016/S0140-6736(10)60484-9 PMC2904878 20609967

