# Supplementary material for: Interpretable machine learning reveals the association of metabolic, inflammatory, and nutritional indices with ischemic stroke complicated by metabolic abnormality clustering
Source: BMC Cardiovasc Disord. 2026 Aug 13;26:1054. doi: 10.1186/s12872-026-06443-y (PMC13749805; doi:10.1186/s12872-026-06443-y)
Supplement: Supplementary file 1 [file 12872_2026_6443_MOESM1_ESM.docx]

Supplementary Table 2 Comparison of baseline clinical characteristics between acute ischemic stroke (AIS) patients with and without MAC in the training set.

| Variable | AIS without MAC  (N = 1,092) | AIS with MAC  (N = 437) | *p*-value |
| --- | --- | --- | --- |
| Gender (Male, n, %) | 617 (57) | 251 (57) | 0.782 |
| Age (years) | 73 (65, 80) | 70 (60, 77) | < 0.001 |
| Time of onset | 5 (2, 24) | 6 (2, 24) | 0.002 |
| HTN (Yes, n, %) | 579 (53) | 348 (80) | < 0.001 |
| DM (Yes, n, %) | 252 (23) | 246 (56) | < 0.001 |
| CHD (Yes, n, %) | 104 (9.5) | 38 (8.7) | 0.684 |
| AF (Yes, n, %) | 246 (23) | 44 (10) | < 0.001 |
| Anticoagulant drugs, n (%) | 22 (2) | 2 (1) | 0.047 |
| APT, n (Yes, %) | 17 (2) | 3 (1) | 0.270 |
| Statin drugs, n (%) | 15 (1) | 3 (1) | 0.388 |
| Smoking (Yes, n, %) | 262 (24) | 118 (27) | 0.244 |
| Drinking (Yes, n, %) | 186 (17) | 84 (19) | 0.347 |
| SBP (mmHg) | 144 (125, 162) | 150 (134, 167) | < 0.001 |
| DBP (mmHg) | 83 (73, 94) | 87 (76, 95) | < 0.001 |
| NIHSS at admission | 5 (2, 10) | 3 (2, 7) | < 0.001 |
| mRS at discharge | 2 (1, 4) | 2 (1, 4) | < 0.001 |
| NIHSS at discharge | 4 (1, 8) | 2 (1, 6) | < 0.001 |
| Na (mmol/L) | 140.0 (138.0, 142.1) | 139.7 (138.1, 141.5) | 0.047 |
| K (mmol/L) | 3.79 (3.50, 4.05) | 3.73 (3.48, 4.00) | 0.032 |
| Cl (mmol/L) | 105.0 (102.4, 107.1) | 103.9 (101.6, 106.1) | < 0.001 |
| WBC (10^9^/L) | 7.40 (5.80, 9.33) | 7.50 (6.10, 9.10) | 0.613 |
| NEU (10^9^/L) | 5.18 (3.87, 7.31) | 5.08 (3.92, 6.65) | 0.372 |
| PLT (10^9^/L) | 183 (141, 227) | 197 (153, 241) | 0.002 |
| HGB (g/L) | 132 (120, 143) | 137 (124, 148) | < 0.001 |
| MON(10^9^/L) | 0.47 (0.36, 0.61) | 0.45 (0.36, 0.61) | 0.944 |
| LYM(10^9^/L) | 1.29 (0.91, 1.80) | 1.50 (1.12, 1.99) | < 0.001 |
| SII | 703 (411, 1,230) | 621 (401, 1,047) | 0.013 |
| SIRI | 1.73 (1.10, 3.26) | 1.41 (1.00, 2.45) | < 0.001 |
| PIV | 313 (184, 612) | 277 (164, 515) | 0.039 |
| NLR | 3.9 (2.4, 6.8) | 3.3 (2.3, 5.0) | < 0.001 |
| dNLR | 2.62 (1.76, 4.38) | 2.32 (1.70, 3.34) | < 0.001 |
| HALP | 38 (26, 57) | 43 (29, 64) | < 0.001 |
| MAR | 0.012 (0.009, 0.016) | 0.011 (0.009, 0.015) | 0.526 |
| FBG (mmol/L) | 6.7 (5.7, 8.8) | 8.6 (6.9, 11.8) | < 0.001 |
| TyG | 8.75 (8.38, 9.14) | 9.71 (9.28, 10.31) | < 0.001 |
| ALT (U/L) | 17 (13, 24) | 20 (14, 28) | < 0.001 |
| AST (U/L) | 24 (20, 30) | 23 (18, 30) | 0.006 |
| TBIL (umol/L) | 12 (9, 16) | 11 (8, 14) | < 0.001 |
| DBIL (umol/L) | 2.70 (1.90, 3.90) | 2.30 (1.40, 3.10) | < 0.001 |
| IBIL (umol/L) | 9.0 (6.3, 12.1) | 8.5 (5.7, 11.5) | 0.023 |
| Crea (umol/L) | 71 (58, 88) | 73 (59, 90) | 0.465 |
| UA (umol/L) | 327 (266, 396) | 354 (287, 428) | < 0.001 |
| UA/Cr ratio | 4.46 (3.67, 5.42) | 4.73 (3.84, 6.01) | < 0.001 |
| ALB (g/L) | 40.2 (37.5, 42.7) | 41.3 (38.1, 43.7) | < 0.001 |
| TC (mmol/L) | 4.79 (4.00, 5.51) | 5.17 (4.24, 6.10) | < 0.001 |
| LDL_C (mmol/L) | 2.77 (2.24, 3.40) | 3.13 (2.53, 3.80) | < 0.001 |
| HDL_C (mmol/L) | 1.37 (1.18, 1.62) | 1.13 (0.95, 1.35) | < 0.001 |
| TG (mmol/L) | 1.12 (0.85, 1.44) | 2.26 (1.81, 3.22) | < 0.001 |
| RC | 0.49 (0.27, 0.75) | 0.74 (0.44, 1.14) | < 0.001 |
| AIP | -0.08 (-0.23, 0.06) | 0.29 (0.16, 0.45) | < 0.001 |
| LCI | 11 (6, 18) | 33 (20, 54) | < 0.001 |
| non_HDL_C (mmol/L) | 3.32 (2.66, 4.03) | 3.98 (3.17, 4.92) | < 0.001 |
| AC | 2.38 (1.86, 3.06) | 3.39 (2.74, 4.17) | < 0.001 |
| CRI_I | 3.38 (2.86, 4.06) | 4.39 (3.74, 5.17) | < 0.001 |
| CRI_II | 2.01 (1.58, 2.58) | 2.74 (2.24, 3.24) | < 0.001 |
| PNI | 47.0 (43.6, 50.6) | 49.2 (45.2, 53.2) | < 0.001 |
| TOAST, n (%) |  |  | < 0.001 |
| CE | 263 (24) | 49 (11) |  |
| LAA | 291 (27) | 140 (32) |  |
| SAO | 491 (45) | 233 (53) |  |
| SOE | 23 (2) | 9 (2) |  |
| SUE | 24 (2) | 6 (1) |  |
