# Supplementary material for: Interpretable machine learning reveals the association of metabolic, inflammatory, and nutritional indices with ischemic stroke complicated by metabolic abnormality clustering
Source: BMC Cardiovasc Disord. 2026 Aug 13;26:1054. doi: 10.1186/s12872-026-06443-y (PMC13749805; doi:10.1186/s12872-026-06443-y)
Supplement: Supplementary file 2 [file 12872_2026_6443_MOESM2_ESM.docx]

Supplementary Table 1 Comparison of variables containing missing values before and after multiple imputation.

| Variable | Missing case (%) | Complete case data | Mice imputed data | *p*-value |
| --- | --- | --- | --- | --- |
| Na | 18 (0.82) | 139.8 (137.9, 141.7) | 139.9 (138.0, 141.8) | 0.407 |
| K | 18 (0.82) | 3.76 (3.50, 4.00) | 3.76 (3.50, 4.02) | 0.828 |
| Cl | 26 (1.19) | 104.3 (101.9, 106.7) | 104.5 (102.0, 106.7) | 0.292 |
| WBC | 41 (1.88) | 7.5 (6.0, 9.3) | 7.5 (6.0, 9.5) | 0.511 |
| NEU | 41 (1.88) | 5.22 (3.94, 7.08) | 5.23 (3.93, 7.25) | 0.663 |
| PLT | 41 (1.88) | 188 (147, 234) | 188 (145, 233) | 0.809 |
| HGB | 41 (1.88) | 133 (122, 145) | 133 (122, 144) | 0.785 |
| MON | 41 (1.88) | 0.46 (0.36, 0.62) | 0.47 (0.36, 0.62) | 0.803 |
| LYM | 41 (1.88) | 1.35 (0.96, 1.83) | 1.35 (0.96, 1.85) | 0.780 |
| ALT | 20 (0.92) | 18 (13, 26) | 18 (13, 26) | 0.722 |
| AST | 20 (0.92) | 24 (19, 30) | 24 (19, 30) | 0.271 |
| TBIL | 20 (0.92) | 11.3 (8.3, 15.2) | 11.4 (8.3, 15.4) | 0.492 |
| DBIL | 22 (1.01) | 2.5 (1.7, 3.5) | 2.6 (1.8, 3.7) | 0.024 |
| IBIL | 20 (0.92) | 8.8 (6.2, 11.9) | 8.8 (6.2, 12.0) | 0.999 |
| Crea | 20 (0.92) | 70.0 (57.8, 86.0) | 71.0 (58.1, 88.0) | 0.212 |
| UA | 20 (0.92) | 336.0 (272.0, 405.0) | 336.0 (273.0, 409.5) | 0.698 |
| ALB | 20 (0.92) | 40.9 (38.1, 43.4) | 40.6 (37.8, 43.3) | 0.222 |
