# Supplementary material for: Interpretable machine learning reveals the association of metabolic, inflammatory, and nutritional indices with ischemic stroke complicated by metabolic abnormality clustering
Source: BMC Cardiovasc Disord. 2026 Aug 13;26:1054. doi: 10.1186/s12872-026-06443-y (PMC13749805; doi:10.1186/s12872-026-06443-y)
Supplement: Supplementary file 3 [file 12872_2026_6443_MOESM3_ESM.docx]

Supplementary Table 4 Comparison of baseline clinical characteristics between acute ischemic stroke (AIS) patients with and without MAC in the validation set.

| Variable | AIS without MAC  (N = 343) | AIS with MAC  (N = 153) | *p*-value |
| --- | --- | --- | --- |
| Gender (Male, n, %) | 202 (59) | 94 (63) | 0.664 |
| Age (years) | 72 (64, 79) | 69 (59, 76) | 0.007 |
| Time of onset | 4 (1, 9) | 6 (2, 24) | 0.002 |
| HTN (Yes, n, %) | 187 (55) | 118 (77) | <0.001 |
| DM (Yes, n, %) | 93 (27) | 100 (65) | < 0.001 |
| CHD (Yes, n, %) | 28 (8) | 16 (10) | 0.510 |
| AF (Yes, n, %) | 66 (19) | 11 (7.0) | 0.001 |
| Anticoagulant drugs, n (%) | 5 (1) | 2 (1) | 1.000 |
| APT, n (Yes, %) | 4 (1) | 1 (1) | 1.000 |
| Statin drugs, n (%) | 4 (1) | 1 (1) | 1.000 |
| Smoking (Yes, n, %) | 76 (22) | 43 (28) | 0.187 |
| Drinking (Yes, n, %) | 72 (21) | 24 (16) | 0.208 |
| SBP (mmHg) | 146 (119, 172) | 149 (125, 173) | 0.190 |
| DBP (mmHg) | 83 (74, 96) | 85 (73, 93) | 0.989 |
| NIHSS at admission | 4 (2, 9.5) | 3 (2, 8) | 0.041 |
| mRS at discharge | 2 (1, 4) | 2 (1, 3) | 0.145 |
| NIHSS at discharge | 3 (1, 7) | 3 (1, 6) | 0.233 |
| Na (mmol/L) | 140.1 (138.3, 142.0) | 139.5 (137.2, 141.4) | 0.007 |
| K (mmol/L) | 3.70 (3.49, 3.96) | 3.64 (3.47, 3.92) | 0.185 |
| Cl (mmol/L) | 104.9 (101.9, 107.2) | 103.8 (101.0, 105.9) | 0.002 |
| WBC (10^9^/L) | 7.4 (6.0, 9.2) | 7.4 (6.0, 9.9) | 0.597 |
| NEU (10^9^/L) | 5.12 (3.92, 6.97) | 5.06 (3.79, 7.10) | 0.888 |
| PLT (10^9^/L) | 184 (145, 227) | 186 (136, 238) | 0.812 |
| HGB (g/L) | 133 (122, 144) | 135 (124, 149) | 0.176 |
| MON(10^9^/L) | 0.46 (0.35, 0.60) | 0.45 (0.35, 0.60) | 0.950 |
| LYM(10^9^/L) | 1.27 (0.92, 1.79) | 1.4 (1.03, 1.98) | 0.018 |
| SII | 716.57 (417.26, 1124.04) | 596.41 (371.45, 1194.54) | 0.117 |
| SIRI | 1.77 (1.11, 3.11) | 1.48 (1.01, 2.53) | 0.099 |
| PIV | 324.5 (183.1, 566.6) | 268.1(157.8, 533.6) | 0.228 |
| NLR | 4.1 (2.5, 6.7) | 3.4 (2.3, 5.5) | 0.052 |
| dNLR | 2.70 (1.79, 4.35) | 2.41 (1.68, 3.64) | 0.072 |
| HALP | 37.78 (26.43, 56.42) | 43.81 (27.86, 64.74) | 0.029 |
| MAR | 0.01 (0.01, 0.02) | 0.01 (0.01, 0.01) | 0.753 |
| FBG (mmol/L) | 6.68 (5.80, 8.71) | 9.40 (7.30, 12.44) | < 0.001 |
| TyG | 8.79 (8.43, 9.13) | 9.73 (9.31, 10.29) | < 0.001 |
| ALT (U/L) | 17 (13, 24) | 21 (14, 28) | 0.011 |
| AST (U/L) | 24 (20, 30) | 23 (19, 29) | 0.140 |
| TBIL (umol/L) | 12.00 (8.45, 16.05) | 11.30 (8.40, 14.60) | 0.190 |
| DBIL (umol/L) | 2.7 (1.9, 3.9) | 2.4 (1.6, 3.1) | < 0.001 |
| IBIL (umol/L) | 9.0 (6.2, 12.4) | 9.2 (6.2, 11.7) | 0.865 |
| Crea (umol/L) | 70.1 (56.0, 87.0) | 75.9 (60.0, 93.6) | 0.016 |
| UA (umol/L) | 319 (264, 401) | 365 (300, 430) | < 0.001 |
| UA/Cr ratio | 4.48 (3.70, 5.70) | 4.79 (3.84, 6.00) | 0.064 |
| ALB (g/L) | 40.6 (37.9, 43.0) | 41.1 (37.1, 43.6) | 0.588 |
| TC (mmol/L) | 4.81 (4.13, 5.58) | 4.79 (3.91, 5.91) | 0.773 |
| LDL_C (mmol/L) | 2.87 (2.28, 3.47) | 2.92 (2.32, 3.43) | 0.711 |
| HDL_C (mmol/L) | 1.38 (1.21, 1.62) | 1.04 (0.93, 1.24) | < 0.001 |
| TG (mmol/L) | 1.14 (0.88, 1.45) | 2.26 (1.75, 3.21) | < 0.001 |
| RC | 0.49 (0.28, 0.76) | 0.67 (0.34, 1.1) | < 0.001 |
| AIP | -0.08 (-0.22, 0.05) | 0.31 (0.18, 0.47) | < 0.001 |
| LCI | 11.49 (6.99, 18.02) | 29.61 (18.21, 51.38) | < 0.001 |
| non_HDL_C (mmol/L) | 3.38 (2.78, 4.06) | 3.68 (2.80, 4.61) | 0.020 |
| AC | 2.53 (1.92, 3.08) | 3.36 (2.60, 4.15) | < 0.001 |
| CRI_I | 3.53 (2.92, 4.08) | 4.36 (3.60, 5.15) | < 0.001 |
| CRI_II | 2.14 (1.62, 2.58) | 2.71 (2.20, 3.14) | < 0.001 |
| PNI | 47.45 (44.00, 50.50) | 47.90 (44.75, 53.65) | 0.119 |
| TOAST, n (%) |  |  | < 0.001 |
| CE | 76 (22) | 12 (8) |  |
| LAA | 93 (27) | 53 (35) |  |
| SAO | 162 (47) | 82 (54) |  |
| SOE | 6 (2) | 5 (3) |  |
| SUE | 6(2) | 1 (1) |  |
