# Supplementary material for: Interpretable machine learning reveals the association of metabolic, inflammatory, and nutritional indices with ischemic stroke complicated by metabolic abnormality clustering
Source: BMC Cardiovasc Disord. 2026 Aug 13;26:1054. doi: 10.1186/s12872-026-06443-y (PMC13749805; doi:10.1186/s12872-026-06443-y)
Supplement: Supplementary file 4 [file 12872_2026_6443_MOESM4_ESM.docx]

Supplementary Table 3 Comparison of baseline clinical characteristics between acute ischemic stroke (AIS) patients with and without MAC in the test set.

| Variable | AIS without MAC  (N = 470) | AIS with MAC  (N = 184) | *p*-value |
| --- | --- | --- | --- |
| Gender (Male, n, %) | 282 (60) | 115 (63) | 0.617 |
| Age (years) | 72 (65, 79) | 69 (59, 76) | < 0.001 |
| Time of onset | 5 (2, 24) | 7 (2, 24) | 0.065 |
| HTN (Yes, n, %) | 243 (52) | 143 (78) | < 0.001 |
| DM (Yes, n, %) | 133 (28) | 99 (54) | < 0.001 |
| CHD (Yes, n, %) | 30 (6) | 17 (9) | 0.270 |
| AF (Yes, n, %) | 75 (16) | 11 (6) | < 0.001 |
| Anticoagulant drugs, n (%) | 8 (2) | 2 (1) | 0.733 |
| APT, n (Yes, %) | 2 (0) | 1 (1) | 1.000 |
| Statin drugs, n (%) | 1 (0) | 1 (1) | 0.484 |
| Smoking (Yes, n, %) | 133 (28) | 64 (35) | 0.126 |
| Drinking (Yes, n, %) | 105 (22) | 37 (20) | 0.605 |
| SBP (mmHg) | 144 (129, 161) | 150 (134, 164) | 0.059 |
| DBP (mmHg) | 83 (72, 94) | 86 (78, 95) | 0.011 |
| NIHSS at admission | 5 (2, 10) | 4 (2, 7) | 0.076 |
| mRS at discharge | 2(1, 4) | 2 (1, 4) | 0.050 |
| NIHSS at discharge | 3 (1, 8) | 3 (1, 5) | 0.065 |
| Na (mmol/L) | 139.8 (138.0, 141.7) | 139.8 (137.5, 141.4) | 0.408 |
| K (mmol/L) | 3.76 (3.50, 4.02) | 3.67 (3.44, 3.98) | 0.058 |
| Cl (mmol/L) | 104.5 (102.0, 106.7) | 103.8 (101.0, 105.8) | 0.008 |
| WBC (10^9^/L) | 7.60 (6.20, 9.60) | 8.05 (6.58, 10.23) | 0.016 |
| NEU (10^9^/L) | 5.38 (4.05, 7.51) | 5.73 (4.22, 7.73) | 0.252 |
| PLT (10^9^/L) | 188 (147, 238) | 187 (149, 230) | 0.782 |
| HGB (g/L) | 133 (122, 143) | 138 (127, 150) | 0.005 |
| MON(10^9^/L) | 0.47 (0.35, 0.61) | 0.49 (0.39, 0.67) | 0.035 |
| LYM(10^9^/L) | 1.30 (0.94, 1.80) | 1.51 (1.03, 2.00) | 0.006 |
| SII | 764 (465, 1,305) | 606 (402, 1,336) | 0.110 |
| SIRI | 1.92 (1.12, 3.49) | 1.74 (1.00, 3.84) | 0.886 |
| PIV | 360 (189, 666) | 321 (175, 707) | 0.755 |
| NLR | 4.2 (2.6, 7.0) | 3.5 (2.2, 6.7) | 0.128 |
| dNLR | 2.77 (1.88, 4.47) | 2.54 (1.69, 4.13) | 0.093 |
| HALP | 38 (26, 56) | 47 (31, 65) | < 0.001 |
| MAR | 0.012 (0.009, 0.015) | 0.012 (0.009, 0.016) | 0.164 |
| FBG (mmol/L) | 6.9 (5.8, 9.4) | 9.0 (7.1, 12.7) | < 0.001 |
| TyG | 8.83 (8.44, 9.26) | 9.74 (9.36, 10.25) | < 0.001 |
| ALT (U/L) | 18 (13, 26) | 21 (15, 29) | 0.003 |
| AST (U/L) | 24 (20, 30) | 22 (18, 28) | < 0.001 |
| TBIL (umol/L) | 11 (8, 16) | 11 (8, 15) | 0.119 |
| DBIL (umol/L) | 2.60 (1.80, 3.70) | 2.40 (1.70, 3.30) | 0.029 |
| IBIL (umol/L) | 9.0 (6.2, 12.5) | 8.5 (6.0, 11.6) | 0.301 |
| Crea (umol/L) | 69 (57, 82) | 72 (60, 95) | 0.006 |
| UA (umol/L) | 332 (264, 399) | 361 (308, 436) | < 0.001 |
| UA/Cr ratio | 4.69 (3.79, 5.71) | 4.96 (3.96, 5.92) | 0.152 |
| ALB (g/L) | 40.8 (37.8, 43.4) | 41.6 (38.8, 44.0) | 0.048 |
| TC (mmol/L) | 4.93 (4.21, 5.80) | 5.08 (4.02, 6.08) | 0.209 |
| LDL_C (mmol/L) | 2.93 (2.36, 3.58) | 3.03 (2.42, 3.67) | 0.316 |
| HDL_C (mmol/L) | 1.38 (1.20, 1.59) | 1.04 (0.91, 1.34) | < 0.001 |
| TG (mmol/L) | 1.18 (0.87, 1.50) | 2.26 (1.79, 3.19) | < 0.001 |
| RC | 0.51 (0.31, 0.81) | 0.81 (0.44, 1.21) | < 0.001 |
| AIP | -0.08 (-0.22, 0.07) | 0.30 (0.18, 0.50) | < 0.001 |
| LCI | 12 (7, 20) | 32 (19, 59) | < 0.001 |
| non_HDL_C (mmol/L) | 3.50 (2.81, 4.30) | 3.97 (3.13, 4.83) | < 0.001 |
| AC | 2.48 (1.93, 3.05) | 3.47 (2.80, 4.42) | < 0.001 |
| CRI_I | 3.48 (2.93, 4.05) | 4.47 (3.80, 5.42) | < 0.001 |
| CRI_II | 2.06 (1.62, 2.58) | 2.78 (2.26, 3.37) | < 0.001 |
| PNI | 47.5 (44.1, 50.9) | 48.8 (45.2, 53.1) | 0.002 |
| TOAST, n (%) |  |  | < 0.001 |
| CE | 83 (18) | 13 (7) |  |
| LAA | 146 (31) | 68 (37) |  |
| SAO | 218 (46) | 99 (54) |  |
| SOE | 14 (3) | 4 (2) |  |
| SUE | 9 (2) | 0 (0) |  |
