# Supplementary material for: Interpretable machine learning reveals the association of metabolic, inflammatory, and nutritional indices with ischemic stroke complicated by metabolic abnormality clustering
Source: BMC Cardiovasc Disord. 2026 Aug 13;26:1054. doi: 10.1186/s12872-026-06443-y (PMC13749805; doi:10.1186/s12872-026-06443-y)
Supplement: Supplementary file 5 [file 12872_2026_6443_MOESM5_ESM.docx]

Supplementary Table 5 Predictive performance of eleven machine learning algorithms in the training cohort.

| Model | Sensitivity | Specificity | PPV | NPV | ACC | Precision | F1-score | AUC |
| --- | --- | --- | --- | --- | --- | --- | --- | --- |
| rf | 1.000 | 1.000 | 1.000 | 1.000 | 1.000 | 1.000 | 1.000 | 1.000 |
| xgbTree | 0.995 | 1.000 | 1.000 | 0.998 | 0.999 | 1.000 | 0.998 | 1.000 |
| rpart | 0.915 | 0.983 | 0.955 | 0.967 | 0.963 | 0.955 | 0.935 | 0.979 |
| earth | 0.833 | 0.954 | 0.879 | 0.935 | 0.920 | 0.879 | 0.855 | 0.970 |
| gbm | 0.696 | 0.997 | 0.990 | 0.891 | 0.911 | 0.990 | 0.817 | 0.962 |
| svmRadial | 0.780 | 0.957 | 0.879 | 0.916 | 0.906 | 0.879 | 0.827 | 0.960 |
| svmLinear | 0.725 | 0.937 | 0.821 | 0.895 | 0.876 | 0.821 | 0.770 | 0.937 |
| glm | 0.730 | 0.924 | 0.794 | 0.895 | 0.869 | 0.794 | 0.760 | 0.939 |
| bayesglm | 0.730 | 0.923 | 0.792 | 0.895 | 0.868 | 0.792 | 0.760 | 0.939 |
| glmnet | 0.728 | 0.924 | 0.793 | 0.895 | 0.868 | 0.793 | 0.759 | 0.939 |
| naive_bayes | 0.801 | 0.884 | 0.734 | 0.917 | 0.860 | 0.734 | 0.766 | 0.927 |

PPV, positive predictive value; NPV, negative predictive value; ACC, accuracy; AUC, area under the receiver operating characteristic curve.
